# GEARing smart environments for pediatric motor rehabilitation

**DOI:** 10.1186/s12984-020-0647-0

**Published:** 2020-02-10

**Authors:** Elena Kokkoni, Effrosyni Mavroudi, Ashkan Zehfroosh, James C. Galloway, Renè Vidal, Jeffrey Heinz, Herbert G. Tanner

**Affiliations:** 1 Department of Bioengineering, University of California, Riverside, CA 92521 USA; 2 Department of Mechanical Engineering, University of Delaware, Newark, DE 19716 USA; 3 Department of Biomedical Engineering and Mathematical Institute for Data Science, Johns Hopkins University, Baltimore, MD 21218 USA; 4 Department of Physical Therapy and Biomechanics and Movement Science Program, University of Delaware, Newark, DE 19713 USA; 5 Department of Linguistics and Institute of Advanced Computational Science, Stony Brook University, Stony Brook, NY 11794 USA

**Keywords:** Body weight support, Human-robot interaction, Activity recognition, Decision making, Pediatric rehabilitation

## Abstract

**Background:**

There is a lack of early (infant) mobility rehabilitation approaches that incorporate natural and complex environments and have the potential to concurrently advance motor, cognitive, and social development. The Grounded Early Adaptive Rehabilitation (GEAR) system is a pediatric learning environment designed to provide motor interventions that are grounded in social theory and can be applied in early life. Within a perceptively complex and behaviorally natural setting, GEAR utilizes novel body-weight support technology and socially-assistive robots to both ease and encourage mobility in young children through play-based, child-robot interaction. This methodology article reports on the development and integration of the different system components and presents preliminary evidence on the feasibility of the system.

**Methods:**

GEAR consists of the physical and cyber components. The physical component includes the playground equipment to enrich the environment, an open-area body weight support (BWS) device to assist children by partially counter-acting gravity, two mobile robots to engage children into motor activity through social interaction, and a synchronized camera network to monitor the sessions. The cyber component consists of the interface to collect human movement and video data, the algorithms to identify the children’s actions from the video stream, and the behavioral models for the child-robot interaction that suggest the most appropriate robot action in support of given motor training goals for the child. The feasibility of both components was assessed via preliminary testing. Three very young children (with and without Down syndrome) used the system in eight sessions within a 4-week period.

**Results:**

All subjects completed the 8-session protocol, participated in all tasks involving the selected objects of the enriched environment, used the BWS device and interacted with the robots in all eight sessions. Action classification algorithms to identify early child behaviors in a complex naturalistic setting were tested and validated using the video data. Decision making algorithms specific to the type of interactions seen in the GEAR system were developed to be used for robot automation.

**Conclusions:**

Preliminary results from this study support the feasibility of both the physical and cyber components of the GEAR system and demonstrate its potential for use in future studies to assess the effects on the co-development of the motor, cognitive, and social systems of very young children with mobility challenges.

## Background

Mobility limitations early in life affect the perceptual, cognitive, and language development of children [1–4]. Mobility training in enriched environments (aka ‘natural environments’ or ‘real world’) can advance experiences, learning, and potentially overall development [5–8].

The timing, type, and dosage of exposure in a learning environment are thought to positively influence development. Exposure during infancy is optimal, as the major developmental milestones and the majority of brain changes occur during the first two years of life [9–11]. Training in enriched environments can lead to experience-dependent brain and behavioral changes; at the same time, high-dose training increases the potential for change [12–14]. In animal studies, environments are enriched with inclined surfaces and objects to promote variability in the motor repertoire. Similarly, these objects have begun to be utilized to study the impact of such environments on typical human behavior and development [15, 16]. In pediatric rehabilitation, however, traditional mobility training does not concurrently address the elements of motor variability, perceptual tasks, and socialization, despite the strong rationale and urgency expressed for involving those elements [8, 17–19].

Self-exploration of an enriched environment is often challenging for young children with developmental delays and mobility issues. One specific challenge for many pediatric populations is the higher effort required to explore. In this methodology paper, we describe the development of the Grounded Early Adaptive Rehabilitation (GEAR) system that aims to alleviate constraints associated with this challenge. Within a physically and socially enriched environment, GEAR utilizes novel body-weight support (BWS) technology and socially-assistive robots to both ease and encourage mobility in young children through play-based, child-robot interaction (CRI). Compared to the state-of-the-art in pediatric rehabilitation, this system innovates by both (a) integrating passive and active elements, and (b) allowing these distinct components to function and interact with each other. The BWS device compensates for a portion of the child’s weight and eases their movement and mobility in an open area. At the same time — and building on the idea that training should not solely focus on the acquisition of motor skills but should rather be grounded in social interaction [17]— mobile robots engage socially with children in structured play activities in ways designed to motivate body movement and open-area exploration. Nesting an infant’s movement and mobility within a physically and socially enriched environment is predicted by embodied development theory to have an impact on the coupled motor-perceptual-cognitive-social development [20–22].

Traditionally, BWS devices are designed to train the single skill of walking and are commonly used over treadmills. Single skill training has difficulty facilitating other activities typically observed in early human development, such as crawling and postural transitions. Moreover, treadmill training with BWS was adapted from adult rehabilitation where it is used to promote motor (re) learning in isolation from enriched environments; this may not be the most suitable training solution for very young children to learn their first functional movements, which requires more general development than simply learning a movement. A notable BWS device exception is the Zero G (Aretech, Ashburn, VA), developed in the last decade to dynamically support various locomotor tasks (i.e., climbing stairs) [23]. This device was used in a pilot pediatric rehabilitation study that combined BWS and motor variability in the training, leading to gains on the motor function of children with cerebral palsy [24]. This device was designed specifically for the clinic– it is single track and non-portable. The BWS device used in our work is part of a new generation of devices designed specifically for open-area, multi-level, real-world mobility (Enliten, LLC, Newark, DE) [25, 26]. The first non-portable version of this series was recently documented in a pilot in-home pediatric rehabilitation study supporting gains in the mobility of a child with spina bifida that were associated with device use [26]. To be clear, the purpose of the GEAR system is to similarly assist infants with learning to use their effective movement and mobility (through the BWS device) as a means to the ends of exploring the environment and interacting socially with the robots. As in typical development, the initial motor-cognitive-social learning will in turn require the infant to continue to learn more advanced movement and mobility strategies.

In GEAR, socially assistive robots have an important role in assisting infants’ learning. Socially assistive robots are different from other assistive robots in that they aim to facilitate close and effective (but not necessarily contact-involving) interactions with the human, not for the sake of substituting for or supporting biomechanical action, but for the purpose of delivering assistance aiming at measurable self-improvement (in the human’s learning, etc.) [27]. In pediatric rehabilitation, specifically, the use of socially assistive robots to promote the social and motor skills of children with disabilities remains limited. One of the few initial applications was robotic therapy for children with social deficits, such as autism spectrum disorder, that mainly focused on the socialization aspect by engaging children in social interactions with the robots [28]. These interactions have even served as catalysts for triggering children’s social interactions with adults [29]. Later studies extended the use of socially assistive robots in motor training paradigms to encourage children’s motor actions mainly through imitation [30–32]. Imitation of a humanoid’s kicking actions was recently documented even in infants, as they both participated in a stationary motor learning paradigm [33]. Current CRI paradigms, however, typically involve a one-dimensional type of interaction; the way robots interact with subjects is always the same. In addition, CRI has not been adequately explored with children younger than two years of age while being engaged in various complex motor tasks, such as those performed during exploration of an enriched environment. In this case, the robots’ adaptation to dynamic human activity can be critical for “guiding” children safely and effectively throughout the environment. Consequently, the goal in the GEAR system was to enable the robots to learn from each interaction, develop personalized behavioral models for each child, and select their own action (eventually in real-time) through feedback received about the child’s evolving motor responses.

**Fig. 1:**
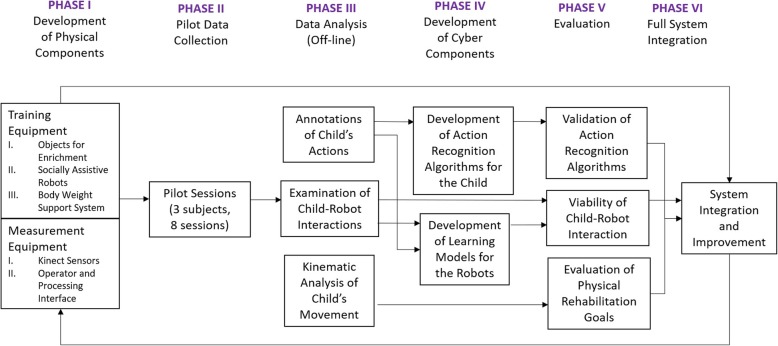
Different phases in the development of the GEAR system

The GEAR system was developed by an interdisciplinary team utilizing both engineering and clinical expertise. This paper describes the different components of the system and provides feasibility results on each component from preliminary testing (Fig. 1).

## Methods

### Description of GEAR as a cyber-physical system

**Fig. 2:**
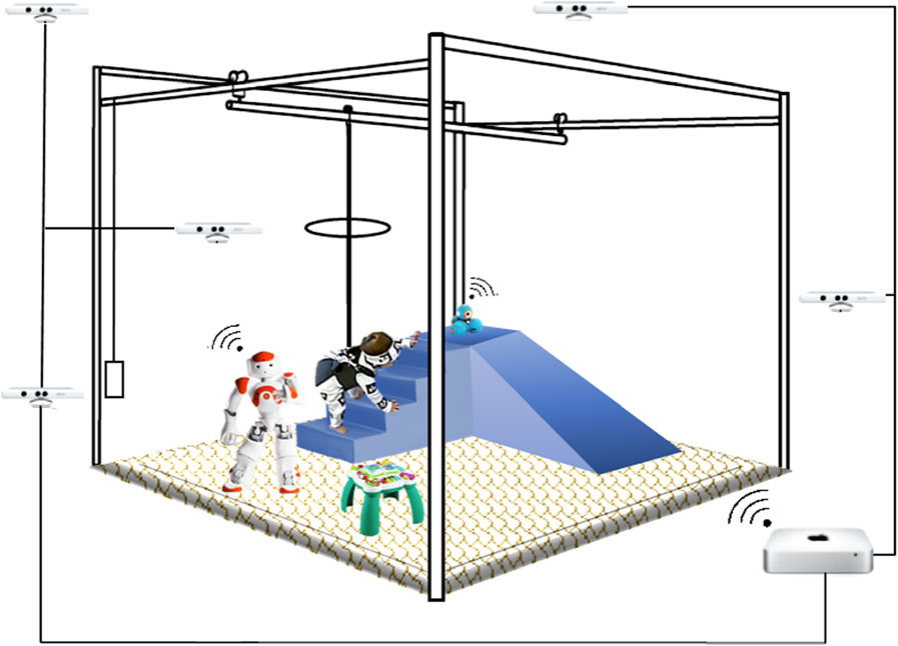
The GEAR environment system concept includes playground equipment, an open-area body weight support device, and socially assistive robots to maximize children’s learning. Kinect sensors, strategically placed around the play area, synchronously collect information about the child’s actions from different angles, and send it to a central server that interprets the scene and instructs the robots

**Fig. 3:**
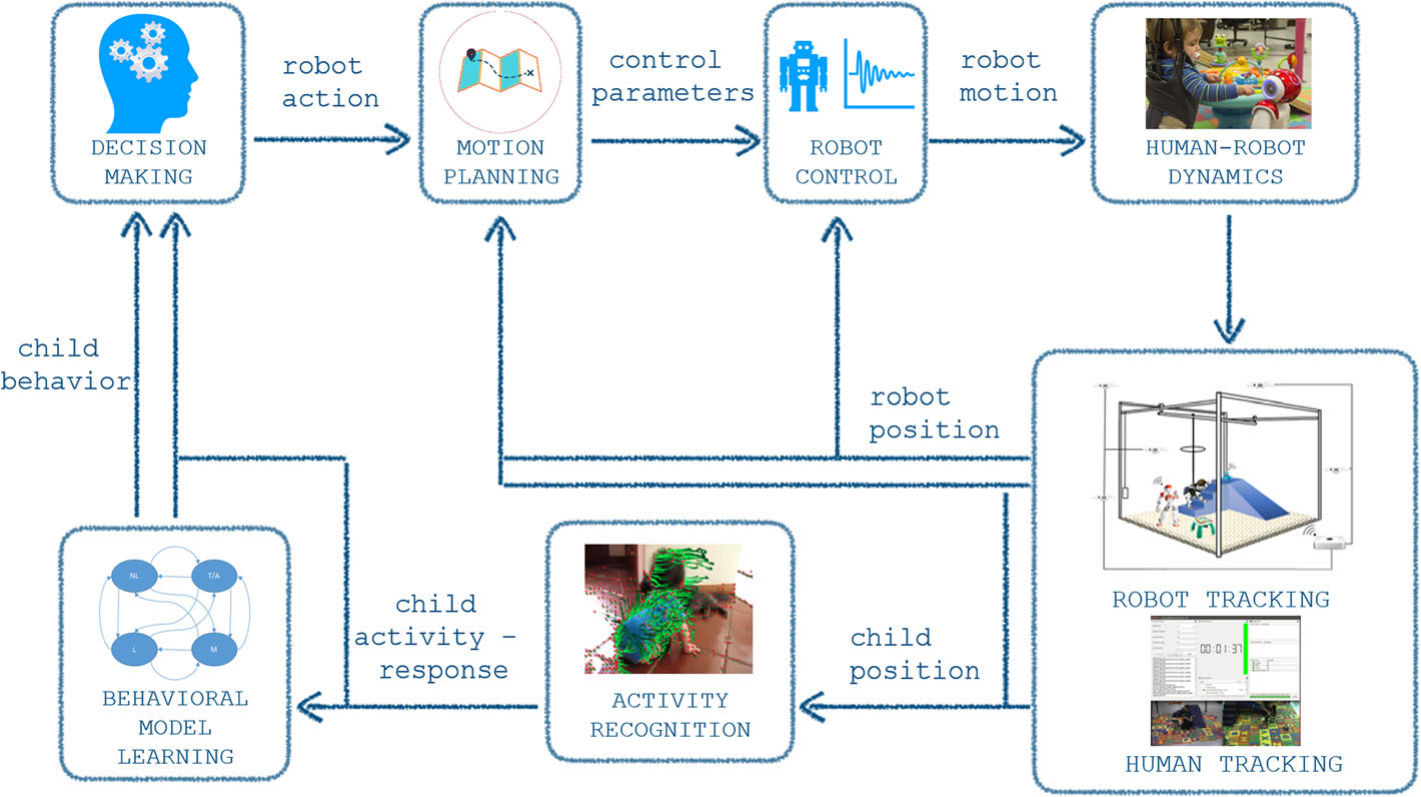
The GEAR system cyber component architecture

GEAR’s physical component includes the playground environment, the BWS device, the robots, and the camera network connected to the main central processing unit which handles data collection (Fig. 2). Its cyber component consists primarily of the software that manages movement and video data collection, the algorithms to identify the children’s actions from the video stream, and the behavioral models for the child-robot interaction that suggest the most appropriate robot action in support of given motor training goals for the child (Fig. 3).

#### The physical component

##### Playground equipment

The GEAR environment was designed to include objects that fit the complexity and novelty features of enrichment so as to induce variability in the children’s motor actions [34]. Figure 2 illustrates the placement of a large foam-padded inclined platform and staircase, as well as a multi-activity high-surface toy. Very young children do not typically encounter these objects in daily life, and especially not before having obtained a certain level of motor ability. Ascending and descending the inclined platform and staircase are novel tasks that afford advanced motor actions, such as crawling and scooting, sometimes even a combination of them [15, 16]. Manipulation of the chest-high multi-activity toy affords practicing transitions such as sit-to-stand, postural actions such as standing, and locomotor actions such as cruising [35]. In addition, the object placement is such to allow open space for spontaneous activity that is not task- or object-specific so that children can practice stepping and safe falling as they learn how to walk [36]. Consequently, even within one session, children have the opportunity to experience various actions that require different levels of balance, coordination, and strength. In the current implementation, the environment is enclosed in a 100 ft^2^ area envelope.

##### BWS device

The BWS device is a patented, recently FDA-registered, commercial device (Oasus™; Enliten, LLC), early versions of which were co-designed by one of the authors. It consists of an overhead support rail structure and a counterweight (Fig. 2). The rail structure consists of two 10 ft-long parallel beams and a perpendicular mobile 10 ft-long beam that gives freedom of horizontal motion to a wearable harness (cf. My Early Steps™ by Little Dundi LLC). Via a system of pulleys and a movable beam, the harness is connected to a counterweight that produces an adjustable vertical force, counteracting gravity. Manipulation of the BWS through variable counterweights passively assists movement in the vertical plane. It must be stressed, however, that the future goal in training with the BWS device is to gradually decrease the amount of BWS so as to avoid the child’s sole reliance to the system for moving around. This particular design allows for practicing a range of motor actions afforded in our enriched environment, including sitting, crawling, standing, walking, climbing, safe falling, and transitions (i.e. sit-to-stand).

##### Socially assistive robots

The selected robots are dynamic, adaptive, and real-time controllable toys, in the role of actuators for the GEAR cyber-physical system. A 58 cm-tall humanoid (NAO™; Aldebaran Robotics) and a small wheeled programmable toy robot (Dash™; Wonder Workshop) are controlled so as to engage the child in imitation and chasing games. The humanoid robot imitates human motor actions such as hand manipulation of the multi-activity toy, sitting, standing, falling, and walking while holding a toy in its hand; The wheeled robot moves horizontally and climbs (mildly) inclined surfaces, reaching a maximum speed on level ground of up to 3.3 ft./sec; making it suitable for chasing games throughout the open area. Dedicated APIs and SDKs for the two robots allow us to establish a limited level of autonomy during these tasks. The robot motion planning strategies that guide the robots in their interaction with the child are derived formally through (discrete) optimal control formulations, in which utility functions capture the intention of keeping the child in motion while performing motor tasks (more on that in the Desicion Making section). Wireless bidirectional communication between the robots and remote devices (e.g., the GEAR processing center and operator interface) is established via Wi-Fi and Bluetooth.

#### The cyber component

##### GEAR user Interface

**Fig. 4:**
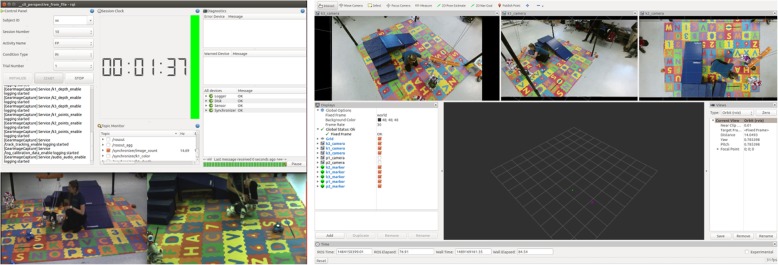
Screenshots of the GEAR interface during a training session

The GEAR processing and operator interface resides in a remote computer workstation and is used for acquisition, processing, transferring, and storage of data from the training sessions. The user and data collection interface has been implemented within the robot operating system (ROS). A graphical user interface provides real-time monitoring of camera feeds and recordings (including video stream, frame-rate, sensor status, etc.) and allows controlled initiation and termination of the recording of particular segments of the training session (Fig. 4). The system records synchronized video streams from a network of five sensors (Kinect® version 2; Microsoft) at 15 frames per second (fps) and tracks the spatial coordinates of AR tags placed on the child’s body. These data inform decision-making for robot action: video is used to train specialized human action classification algorithms, which together with real-time measurements of robot(s) and child’s position can dictate what is hypothesized as the most effective course of action for the robot in order to entice the child’s desired response.

##### Action classification

The goal of the action classification component is essential for identifying the sequence of the child’s motor actions from the video data recorded during the sessions. Its output will eventually be utilized to close the control loop with the robot and let it plan its future actions completely autonomously. Certain challenges are associated with this component. Recognizing sequences of actions performed by humans in long untrimmed videos (and determining their temporal boundaries) is an active research field [37, 38]. In GEAR, this task is further complicated due to the existence of multiple concurrently acting entities (the child, robots, family members, physical therapist, etc.), whereas the activities of interest are only those of the child. Occlusions are also frequent, either due to the GEAR system apparatus (e.g., BWS device, playground equipment) or due to the researcher/robots interacting with the child. In the next section, we assess the ability of widely used classification algorithms to identifying the child’s activity in this complex setup.

##### Decision making

This component refers to an algorithmic framework for choosing an appropriate action or maneuver for the robot(s) expected to trigger a desired motor response from the child during play-based interaction (e.g. in imitation or chasing games). For example, if the child spends significant time in a sedentary mode (i.e. sitting) the robot(s) may engage in maneuvers (selected to indicate intention to initiate chasing games) that have empirically high likelihood of mobilizing the child. To predict human intention, various Markovian models have been successfully applied in other human-robot interaction applications, such as the Partially Observable Markov Decision Process (POMDP) and the Mixed Observability Markov Decision Process (MOMDP). In our paradigm, we propose a fairly standard and simple model that takes the form of a Markov decision process (MDP). While an MDP may be lacking in mathematical sophistication and descriptive power, it compensates in terms of analytical and computational expediency, yielding a model capable of capturing salient attributes of the behavior of interest with a smaller number of tunable parameters.

**Fig. 5:**
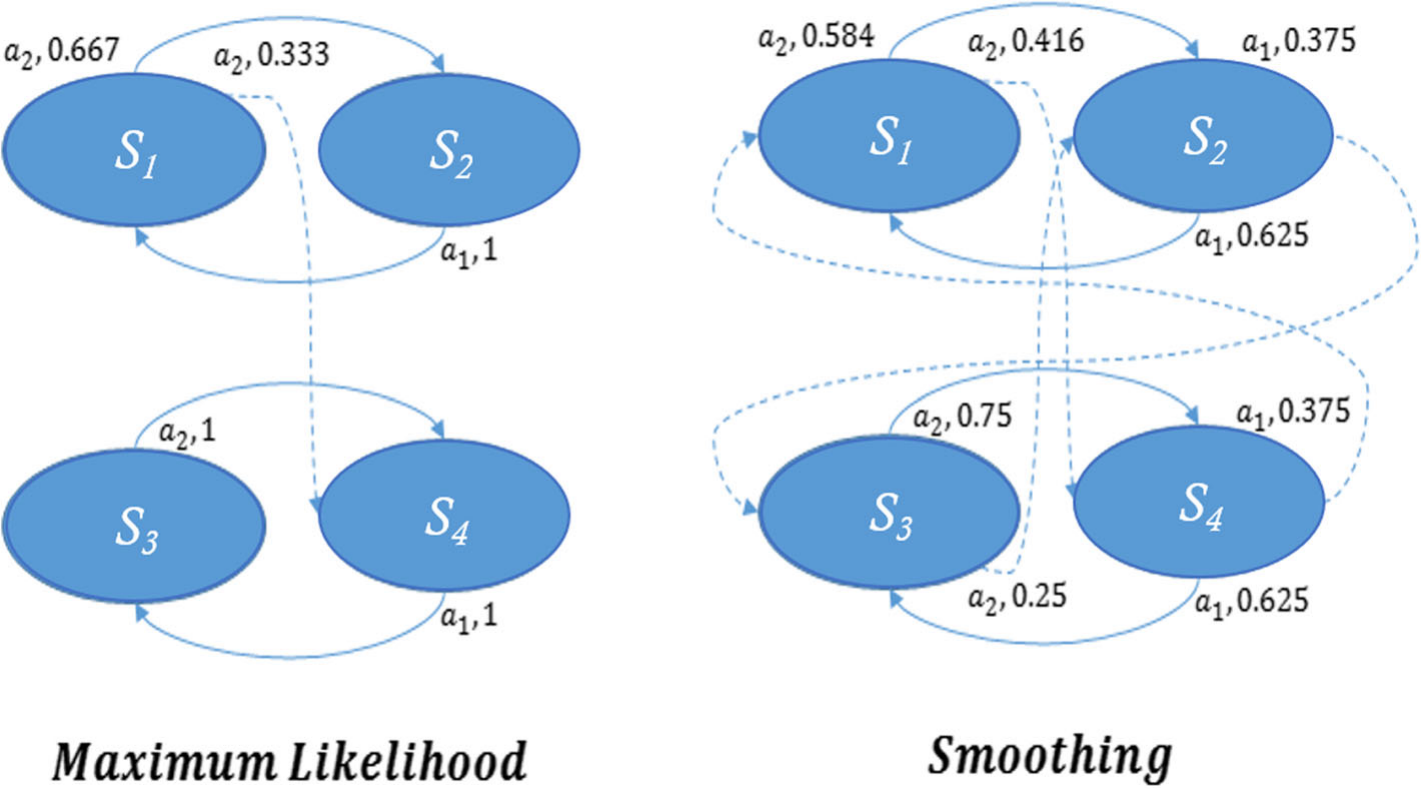
Comparison between the application of maximum likelihood (left) and smoothing (right) for estimating transition probabilities out of small data sets. Smoothing assigns small but nonzero probabilities to events that have not (yet) been observed, acknowledging the fact that the data set may be small and sparse

The MDP that is chosen to model CRI during a chasing game contains states representing possible “modes of operation” for the child. The action set for this model consists of the different options the robot can utilize in order to motivate the child into motor activity and transition among those states. In other words, transitions in this MDP will express the child’s reactions to the robot’s actions. However, a challenge in using the robot’s actions as input is that we do not know a priori how each child will react to the robot’s maneuvers. If the probabilities for the transitions between the states of that MDP were known, then standard methods (e.g. value-iteration, Q-learning, etc) could be used directly to optimally select control policies for the robot during their interactions with the child. Overcoming this challenge necessitates the application of a particular, specialized machine learning technique that identifies a model of behavior for each individual child, based on a very limited set of (supervised) CRI observations. Due to the sparsity of training data in our application, current mainstream machine learning methods can face problems. Techniques designed to address training data sparsity have appeared in early natural language processing –one of them is known as smoothing (Fig. 5) [39]. Smoothing acknowledges the fact that data are sparse, and an event not observed is not necessarily impossible. More information on the specific MDP model employed in GEAR using the smoothing technique is described in the next section.

### Development of the experimental Testbed

#### Human data collection and analysis

Three young subjects participated in eight sessions that took place twice a week for four weeks. Subject 1 was 24 months old and diagnosed with Down syndrome. Subjects 2 and 3 were typically developing and were 11 and 10 months old, respectively. All subjects had acquired similar skills by that age; they were able to sit and crawl independently but not stand and walk without support. The difference in the age of subject 1 was not surprising as very young children with Down syndrome present significant delays and take about twice the time to achieve the onset of motor milestones [40, 41]. Even at that stage, however, the quality and quantity of their motor actions and their perceptual-motor coupling abilities are poor compared to their typically developing peers [42]. Previous research suggests training infants with Down syndrome in early perceptual-motor competencies to address these issues (i.e. encouraging eye gaze and joint attention to objects, people and the environment rather than isolating and training specific motor components) [42, 43]; thus, making subject 1 a suitable candidate for trying the GEAR system. Parents of the enrolled children provided written informed consent for study participation and for pictures to be used in research publications.

**Fig. 6:**
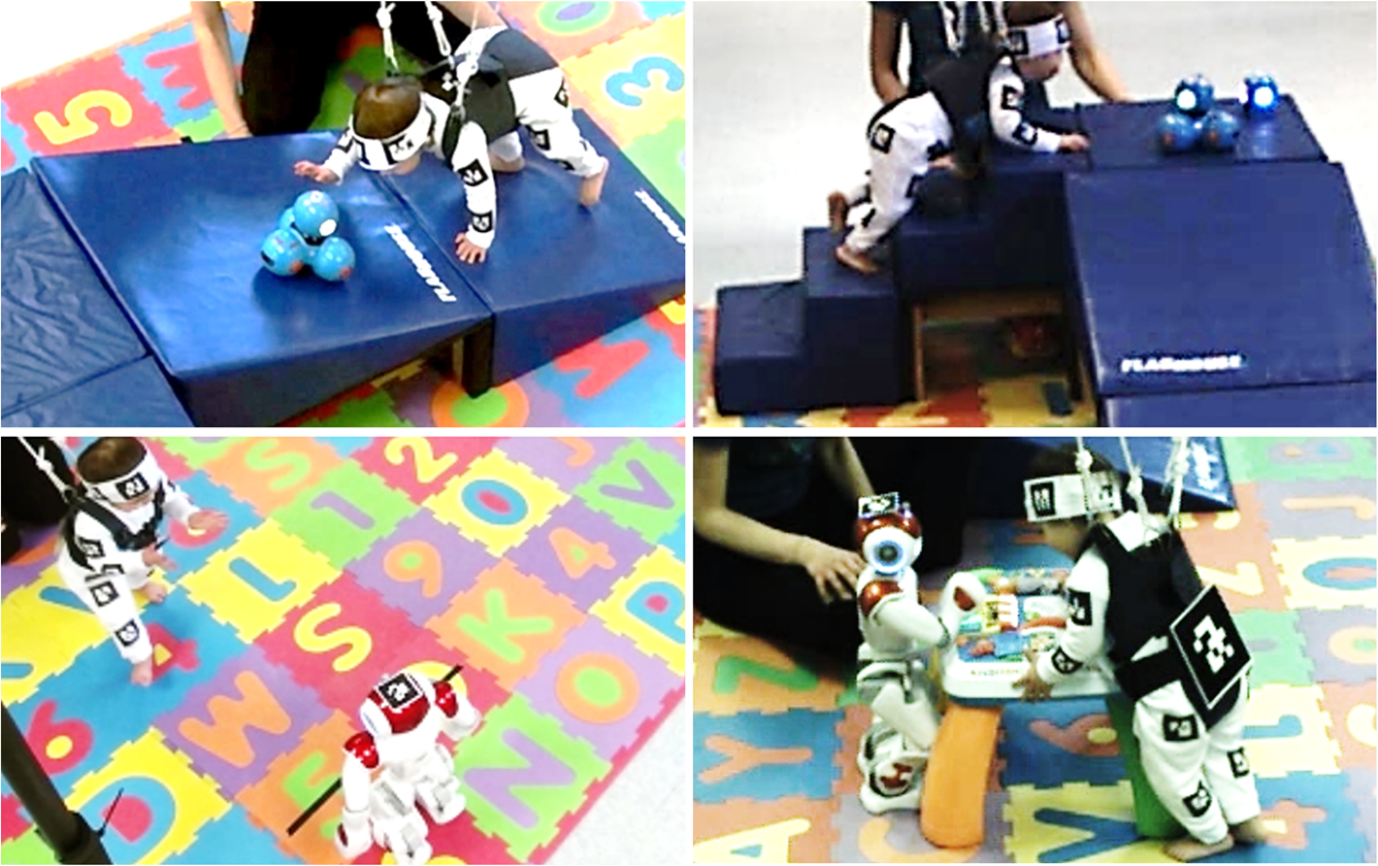
Snapshots of a child within the GEAR system. The child, supported by the device, performs various and complex motor actions and interacts with the robots during exploration and manipulation of the objects of the enriched environment

Each session lasted for about an hour. Within each session, specified chunks of time were allocated to the children to perform motor tasks involving each object of the environment and while interacting with the robots (Fig. 6). These tasks involved: ascending the inclined platform and staircase while chasing the robot Dash (one ascending trial on each object); standing near, and around, a table-toy while manipulating the top surface together with robot NAO (one 3-min trial); crawling and walking on a flat padded surface towards robots Dash and NAO respectively (one trial each); and exhibiting spontaneous mobility while chasing and interacting with both robots in free-play mode (one 3-min trial). These tasks were performed under two conditions; with and without the assistance from the BWS device. The order of the tasks was standardized across sessions and subjects. The order of conditions alternated across sessions for each subject. The BWS ranged from 20 to 60% of the child’s body weight, keeping lower support for movement in the horizontal (i.e., crawling horizontally) and higher support for movement in the vertical plane (i.e., standing next to the table-toy). All sessions were video recorded.

Robots were remotely controlled by a human operator, with the explicit goal to keep the child engaged in the motor task activity through social interaction with the robots. During the chasing games, the distance between the child and the robot was varied while the robots made sounds (playing songs or producing animal sounds) to attract the child’s attention. The initial goal for the robots was to purposefully close their distance from the child when the latter is not engaging in the desired activity to increase the possibility for the child to initiate an approach; based on previous research supporting that crawling infants are more likely to travel to nearby than to distal objects [44, 45]. Then, the goal was for the robots to retreat when the child starts chasing them, and in this way “steer” the latter along a desired direction (this motion control strategy will be referred to as the “regular policy”—more details are offered in the Decision Making section that follows). We utilized the following indicators of interaction between the child and the robot:

- Visual attention to robot: The number of times (per minute) the child looked at the robot during the free play trial.
- Moving towards the robot: The number of times (per minute) the child moved or followed the robot during the free play trial.
- Ascending completion: The number of times each child made a full ascend while following the robot (on platform) or moving towards the robot on top (of staircase).

Our hypothesis was that the majority of children would be engaged in our paradigm through interacting with the robots and moving in the environment in every session.

#### Action classification algorithms

Data from the human subject sessions were used to determine what action classification algorithms would be most effective for classifying the child’s following key motor activities: (i) crawling, (ii) sitting, (iii) standing, and (iv) walking. Assuming that the temporal boundaries of actions were known, the goal was to classify short video segments containing a single action performed by the child into one out of these four categories (a video segment is therefore referred to as video henceforth). The five camera sensors were strategically placed around the scene so that the chance of the child not being visible in all views to be very small –implied is also here the fact that not all five synchronized video streams may feature the child. The system should predict the child’s action by exploiting these views with no a priori knowledge of which ones contains occlusions. The system should also be able to predict the child’s action despite the presence of other moving entities. The two main stages of our pilot action classification component were (a) video representation, and (b) classification.

**Fig. 7:**
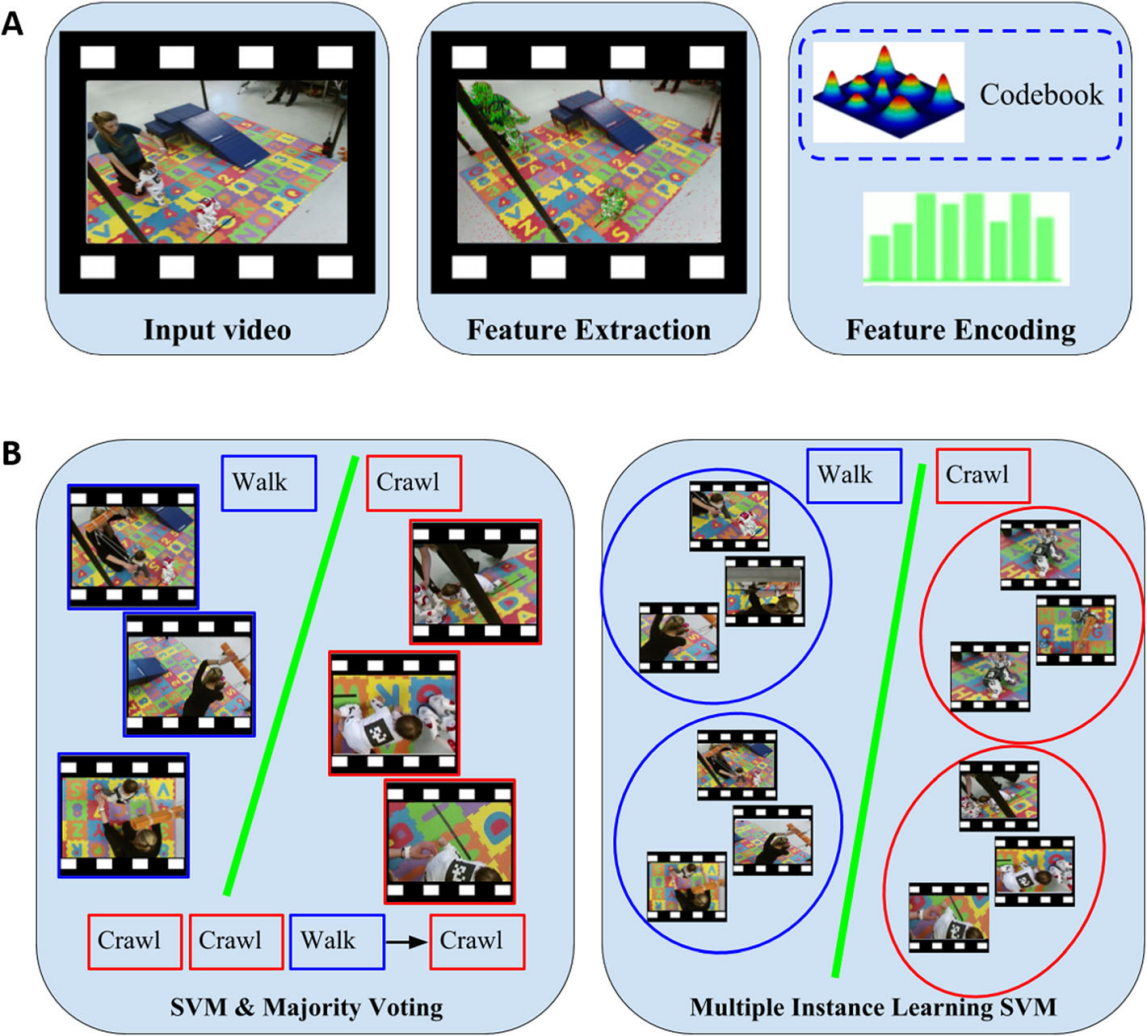
**a.** Overview of video representation framework. **b.** The two approaches for action classification: SVM with Majority Voting fusion (left), Multiple Instance Learning SVM (right). For illustration purposes, we assume three views per action instance. Frames are cropped to focus on the child

- Video Representation: A high dimensional video needs to be described by a more compact, and ideally discriminative, representation. We used the improved dense trajectories (IDT) features [46], one of the most successful hand-crafted features for video action classification. Given a video, the IDT method extracts a set of spatially dense and temporally short trajectories that track moving points for a short period for time (i.e., a second) and then associates a descriptor with each one of the trajectories. A descriptor is a vector which describes the shape of the trajectory, as well as the appearance and movement of a small spatio-temporal tube along the trajectory. After extracting a large number of trajectories and associating a descriptor with each one of them, we proceeded by aggregating them into a single vector describing the video. This was done through a dictionary of codewords, e.g., by assigning each descriptor to its closest codeword and counting how many times each codeword appeared in the video. In particular, we used a Fisher Vector encoding [46], that has been successfully used in the literature in combination with the IDT features. Our video description pipeline is summarized in Fig. 7a.
- Classification: Machine learning techniques were used to predict the label of each action instance performed by the child given the five feature vectors, each one describing the video from one of the five cameras. To train the classifier we used a subset of the recorded action instances viewed by the five cameras, along with the action label of each action instance. Currently, these labels were provided by humans manually annotating each time frame of the five synchronized video streams with an action label as long as the action was clearly visible from at least one camera at this time frame. We explored two approaches to action classification that exploit the availability of five views. In the first approach, during training, the annotated action label of each action instance is transferred to each one of the five corresponding video feeds. Note that this step can inadvertently introduce errors, since not all views may showcase the labeled action, for example, due to some occlusion. A linear support vector machine (SVM) is then trained on the feature vectors extracted from those training videos. The SVM classifier predicts one action label for each view, and the final label for the action instance is derived by a majority voting (MV) late-fusion method —basically assigning the label that was predicted in the majority of the camera views. We refer to this approach as support vector machine with majority voting (SVM-MV). The second approach is based on multiple instance learning SVM classification. Here each training and testing sample is considered to be a bag of videos, and to each such bag, one action label is associated. That is, all five recorded videos of a particular action instance are grouped into one bag. Then, multiple instance learning (MIL) —a weakly supervised learning method— leverages these bag-arranged training data to learn how to classify similar bags during testing, using an MIL extension of SVMs, intuitively referred to as multiple instance support vector machine (MI-SVM) [47]. This method avoids voting schemes for fusing the output of independent classifiers (Fig. 7b).

The proposed action classification framework was evaluated by using the per time frame action labels provided by annotators to determine the temporal boundaries (start and end time frame) of each instance of a single action of interest performed by the child. Using these temporal boundaries, we collected short videos from five cameras capturing these action instances. This preprocessing step yielded 166, 228, 406 and 248 unique action instances for the crawling, sitting, standing and walking action classes, respectively. These action instances were then randomly split into five training and testing sample sets, with each training sample set containing 80% of the recorded action instances in each class, and keeping the rest for testing. This process produced five splits with 840 training instances and 208 testing instances each. Note that since each action instance is recorded by five cameras, these numbers translate to 4200 videos used in training and 1040 videos used in testing. The two action classification approaches were evaluated, measuring performance in terms of the average action instance classification accuracy —in other words, the percentage of correctly classified testing action instances, averaged over the five random splits. We hypothesized that the MI-SVM approach would be more accurate than SVM-MV, as it would result in a larger percentage of correctly classified testing action instances, by better modeling the complementary information captured in the multiple views.

#### Decision making algorithms

**Fig. 8:**
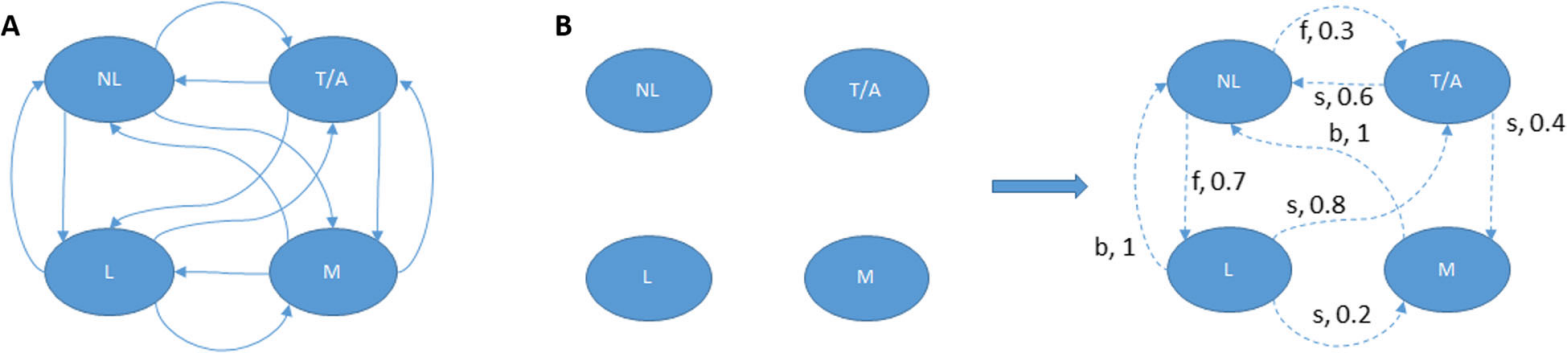
**a.** The MDP model for CRI. Each of the arrows can be labeled by actions with its corresponding transition probabilities. **b.** The initial MDP (left), and the updated MDP after observing some transitions (right)

The goal in this case was to develop and validate an MDP model based on observations from our preliminary testing. An instantiation of the proposed MDP model for the case of a simple chasing game between robot and child is shown in Fig. 8. In this MDP, the action set (robot’s action) is defined as a set {f; s; b}, with *f* representing a decision for the robot to move “forward” and toward the child, *s* associated with staying stationary or turning around while keeping the same distance to the child, and *b* representing a robot command to retreat facing the child. Any transition arrows in Fig. 8a can be labeled by these actions with different probabilities. The states in this discrete model are {NL; L; T/A; M}; with *NL* representing that the child is not looking at the robot, *L* stands for the situation when the child is looking at the robot but not following the robot, T/A represents that the child is touching the robot or is excited (e.g. clapping) by observing the robot’s action, and M stands for the circumstance when the child is following the robot. When the child makes transition to T/A or M, it is a success for the robot. Positive utility is assigned to these favorable states, and negative or zero utility is assigned to the remaining ones. In fact, we assigned {0,0,1,2} utility for states respectively.

The MDP model is originally initialized with no transitions and each state being isolated (Fig. 8b-left). As soon as we start to observe reactions of the child to the robot’s actions, the model is updated. Specifically, each time the child makes a transition from one state to another (as a response to one of the robot’s action), the MDP is updated by adding the transition arrow with its estimated probability (an example of this process is shown in Fig. 8b-right). For example, if by doing action s in state L five times, we have observed that 4 times the child made transition to state T/A, and 1 time the child made transition to state M, then the corresponding transition arrows with their probabilities are shown in Fig. 8b-right (Maximum likelihood is used in this example). This process will in principle continue until the evolving MDP converges to some true hypothesized probabilistic model of this particular CRI [48]. Simulation results with the aforementioned model have so far supported the hypothesis that smoothing can be advantageous as a learning mechanism for populating the unknown parameters of this MDP [48].

In order to evaluate the effectiveness of the above method, we utilized a portion of the free-play mode data observed in regular sessions (by “regular” here we indicate the robot behavior under the direct control of a human operator) to estimate transition probabilities, and then used the mathematically evaluated optimal policy (based on those estimated transition probabilities) to collect a portion of new data for the same task. We then compared the robot’s performance in a regular session to its performance in a session where the computed optimal policy was used. More specifically, we computed the absolute change in accumulated utilities (normalized by time) from the first (session 1) and the last session (session 8) for each subject that were completed within a time window of four weeks. The regular policy was used in both sessions 1 and 8 for both subjects 1 and 2. For subject 3, the regular policy was used in session 1 and the optimal policy was used in session 8. If the absolute change in utilities between sessions of subject 3, where the policy was different in the two sessions, is larger than the change seen in subjects 2 and 3, where the policy remained the same, then this would indicate that our hypothesis regarding the MDP model being appropriate for the proposed paradigm, is valid.

### Hypotheses

The goal of the pilot experiments was to assess the feasibility of GEAR’s both *physical* and *cyber* components. In the first case, we explored the viability of CRI and the children’s participation in the complex motor tasks. We hypothesized that at least two of the subjects will:

H1.1 Direct their visual attention to the robots in the free-play mode at all sessions.

H1.2 Initiate proximity actions (move towards the robot) in the free-play mode at all sessions.

H1.3 Follow the robot on the inclined platform and staircase and will complete at least half of the ascends throughout the study.

In the case of cyber component, we assessed the action classification algorithms and the MDP model. The following hypotheses were formulated:

H2.1 The MI-SVM action classification approach will outperform SVM-MV by providing larger percentages of correctly classified testing action instances.

H2.2 The absolute change in utilities between sessions of subject 3, where the policy was different in the two sessions, is larger than the change seen in subjects 2 and 3, where the policy remained the same.

## Results

### Feasibility of physical component

All subjects completed the 8-session protocol, participated in all tasks involving the selected objects of the enriched environment, used the BWS device and interacted with the robots in all eight sessions. When assisted by the BWS device, all subjects were able to perform motor actions that were beyond their level of capability without assistance by an adult, such as independent steps and bouncing. No adverse events were reported.

**Fig. 9:**
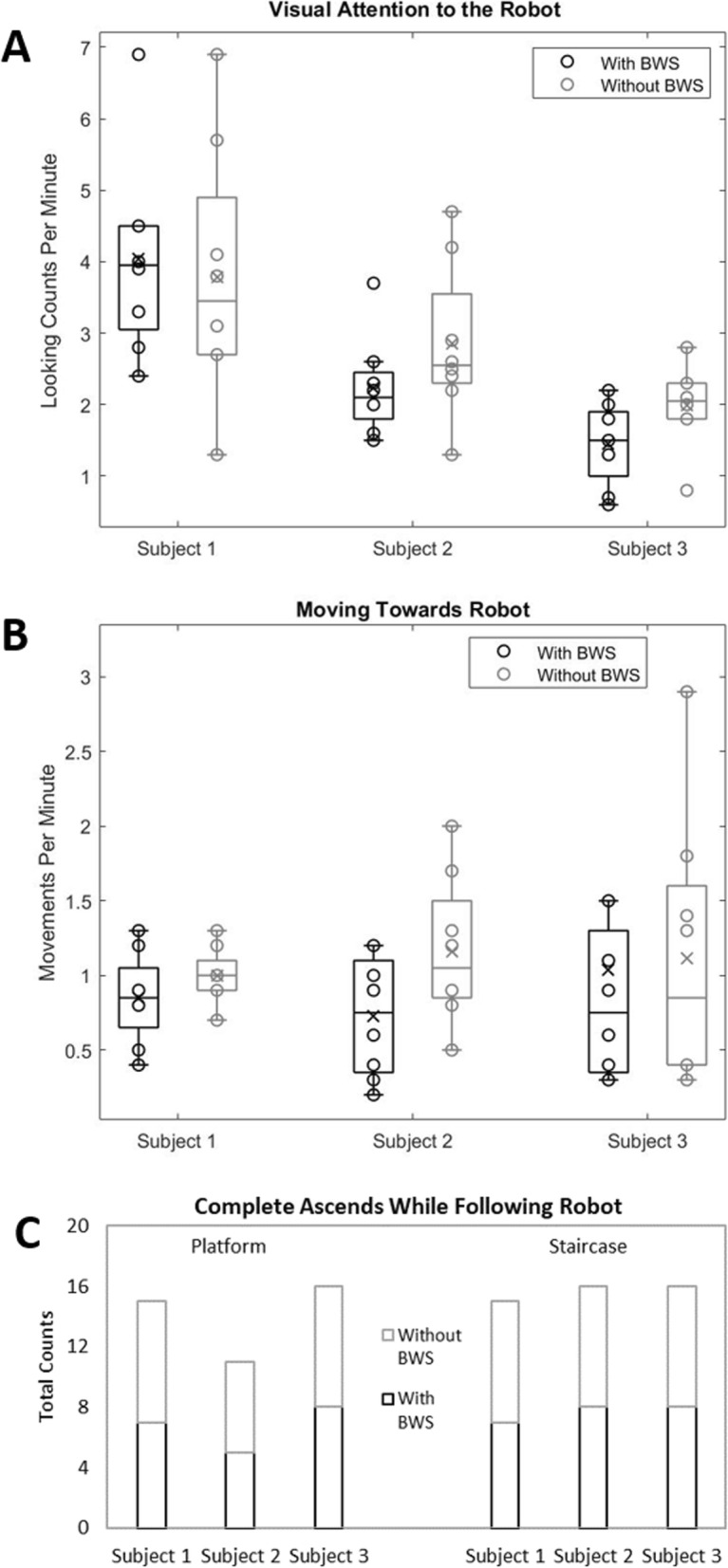
Box Plots depicting number of looking instances per minute (**a)** and number of movements the child initiated towards the robot (**b**) from all sessions. The center box lines represent the median and the box edges the 25th and 75th percentiles. The whiskers show the range up to 1.5 times the interquartile range. **c.** Total number of completed ascending trials on the platform and staircase while following the robot

Figure 9a shows that visual attention to the robots was evident in all sessions. Interestingly, we noticed that visual interest was evident especially during unexpected events; for example, each time robot NAO was falling down and trying to get back on its feet. Overall, subject 1 (child with Down syndrome) demonstrated more interest in looking at the robots than his typically developing peers. The children’s interest in closing the distance between themselves and the robots was also shown in all sessions by all subjects. Figure 9b shows the number of moves the children initiated towards the robots. Furthermore, all children demonstrated great success in completing climbing tasks while following the robots. Figure 9c shows the accumulated complete ascends from all sessions on the platform and the staircase. Lastly, we observed, to our surprise, instances where subjects adapted their motor actions to aid the robots fulfill their goal in the task. For example, when the wheeled robot had trouble ascending the inclined platform, the children would gently push the robot to “help” it. In other occasions where robot NAO was experiencing difficulties while manipulating table-toy interfaces, the child would push the robot’s arm down to activate the buttons on the table-toy.

### Feasibility of cyber component

The action classification results are reported in Table 1 and indicate that for our particular application MI-SVM outperforms SVM-MV. This result validates our hypothesis since SVM-MV uses each recorded video independently for training, by associating with it a ground action label, although the action might not be visible from the viewpoint that the video was captured, while the MI-SVM exploits the complementary information captured in all five views. It is worth noting the variability in action classification performance between different random splits, which showcases not only that different subjects perform the same action in different ways, but also that in an enriched complex environment the same subject can perform the same action differently across different sessions.

**Table 1:** Action classification results. Quantitative comparison of the two classification approaches: SVM-MV and MI-SVM. We report the percentage of correctly classified action instances for each testing set for five random training/test splits as well as the average accuracy over splits

	SVM-MV Accuracy (%)	MI-SVM Accuracy (%)
Split 1	62.02	**70.67**
Split 2	66.35	**68.75**
Split 3	71.15	**73.08**
Split 4	**69.71**	69.23
Split 5	74.52	**80.29**
Avg	68.75	**72.40**

**Fig. 10:**
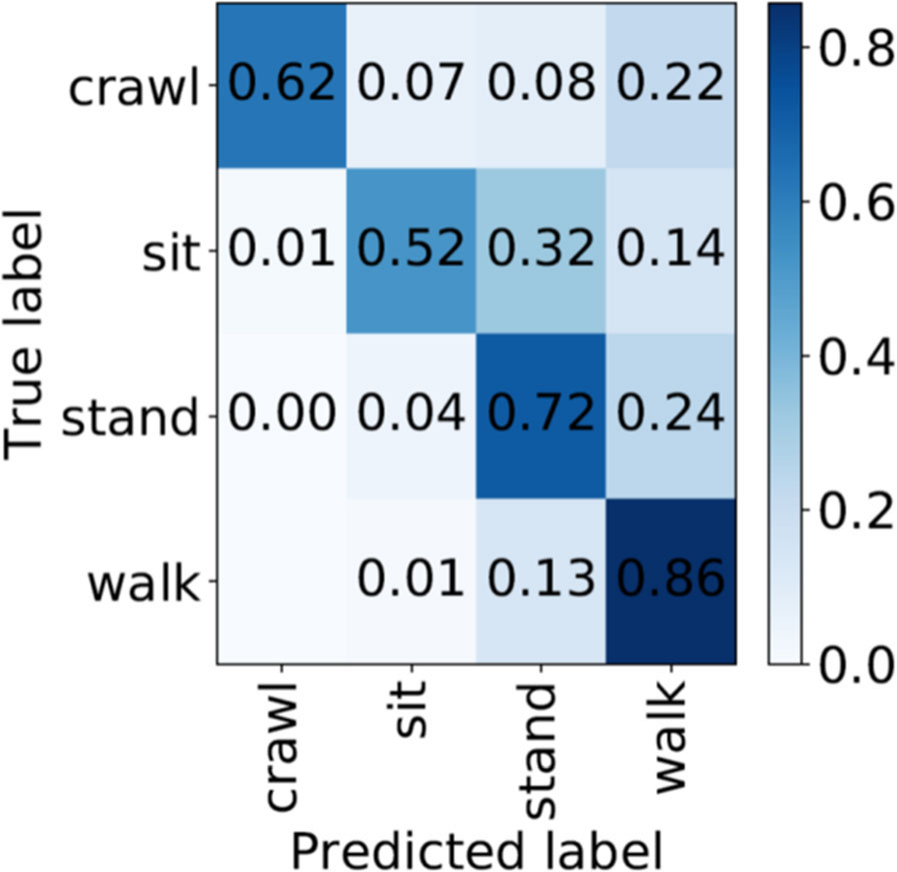
Action classification results using the MI-SVM classification approach. Diagonal entries of confusion matrix show the percentage of correctly classified action instances per action class with respect to ground truth annotations. Results are averaged over five random training/testing splits

Figure 10 shows the average confusion matrix for over five random splits. Our results indicate that MI-SVM can correctly classify most of the walking action instances. The algorithm occasionally confuses standing with walking (i.e. it labels on average ~ 24% of the standing action instances as walking), which may be attributed to instances of walking being very short, e.g. just a single step. A possible reason for occasional failure to distinguish sitting from standing (in ~ 32% of the cases) could be due to the features used: since sitting and standing are static actions, the trajectory-based (and thus motion-dependent) features might not be discriminative enough. We plan to experiment with alternative feature extraction methods to better describe static actions.

**Fig. 11:**
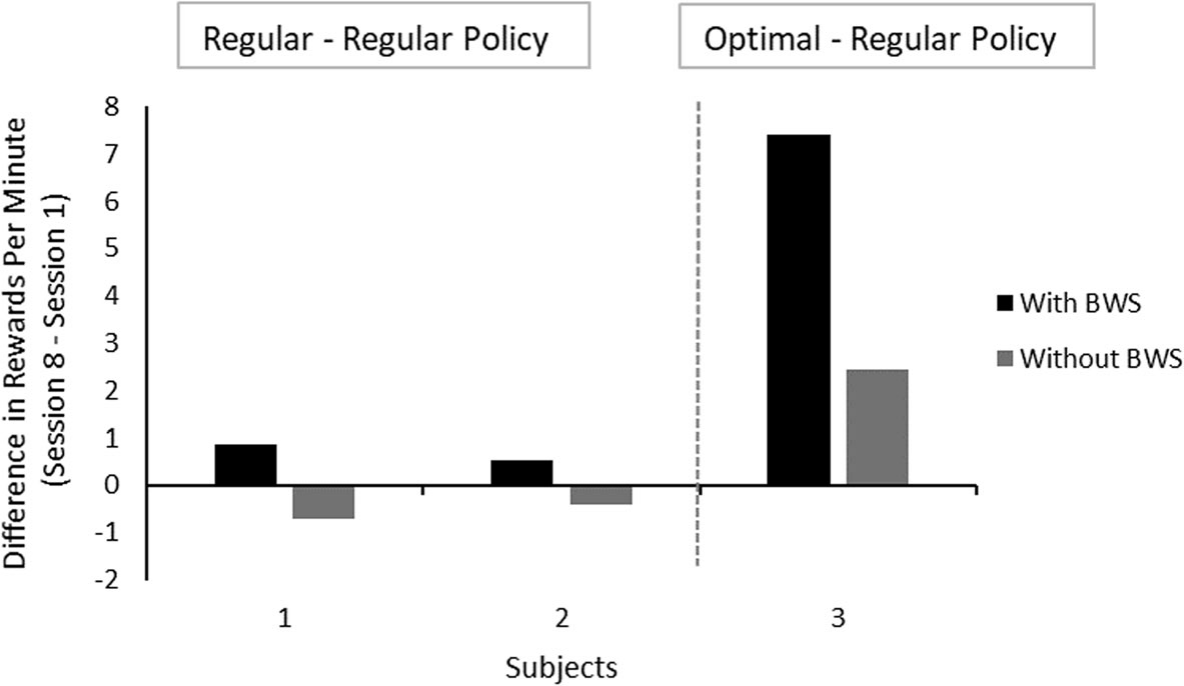
Difference in rewards using the regular (subjects 1 & 2) and optimal policy (subject 3) between the first and the last session. There was a noticeable difference in subject 3 compared to the other two subjects where the performance remained relatively similar

With respect to robot motion planning, autonomy, and deliberative decision making in the context of CRI for motor rehabilitation, difference in utilities in session 8 from session 1 for each subject are shown in Fig. 11. Utilities were similar in the two sessions for subjects 1 and 2 but were very different for subject 3. In addition, it appears as if this difference was bigger when the subject was assisted by the BWS device. The data set is prohibitively small to allow any statistically significant distinctions at this point; at a minimum, however, the observed data do not seem to disprove the hypothesis that improvement in the performance of the robot will occur by using the optimal policy. Nevertheless, these preliminary data encourage pursuing this research approach in the future.

## Discussion

GEAR is a novel robot-assisted learning environment that has shown promise for potential use in pediatric rehabilitation, and this methodology article reports on its design while offering some evidence on the feasibility of the approach. In its pilot implementation, GEAR takes the form of an assisted (through the BWS device) playground that allows complex and rich interaction between children, toys, and robots. Concurrently, a visual sensor network functions with a dual purpose: to record data for assessment of rehabilitation outcomes, and for providing the framework to achieve (real-time) feedback for the automation system to guide the robot in support of these rehabilitation objectives in the future. The initial realization of this combined sensing and computation framework has shown promise with (a) preliminary evidence of affording exploration by three children, and (b) data suggesting the viability of the framework for early CRI.

All subjects interacted with the robots in different quantities. Visual attention was directed from all subjects towards the robots, and especially during surprising events. Increased interest in unexpected events has been previously reported in CRI with older children [49]. In addition, subjects were keen to reduce their proximity distance with the robots, thus maximizing the possibility for close interactions [50]. More importantly, they followed the robots even during complex motor tasks, showing the great potential for use of the robots for activities in enriched environments. Lastly, we observed very young subjects adapting their motor actions to assist robots complete their goal. Human behavioral adaptation in order to assist the robots in performing their tasks has been previously documented in adult human-robot interaction [51], but, to our knowledge, this is the first time that such a human behavioral response is reported in the context of early CRI. Future group studies with the GEAR system will further examine and potentially validate these preliminary but important observations.

The realization, implementation and integration of the engineering components of this system was proven feasible and promising for future extensions and out-of-the-lab implementation. The main challenge that the current action classification framework had to face was the fact that the video representation was obtained from the entire scene and could be affected by multiple people or robots performing some of the actions of interest. Future work will explore ways to better handle the complexity of the scene, for instance detecting the location of the child and focusing on video parts localizing around the child, eliminating the effect of multiple acting entities. Our ultimate goal is to integrate a temporal model that will reason about action durations and transitions, in order to be able to classify the actions performed by the child during its interaction with the robot in a continuous video stream.

Future work should also address certain limitations of this feasibility study. One limitation was that we did not employ eye-tracking technology to measure visual fixations. Visual attention was assessed from the video recordings through an annotation process, a method traditionally used in developmental/behavioral research. Although employing eye tracking technology to measure visual fixations would be ideal, we were skeptical about adding an extra device (e.g., head mounted eye tracker) to the ones already being used in our paradigm, as this might overwhelm the young children. In addition, extracting gaze information from the humanoid’s embedded camera was considered inadequate in our case as the children also interacted with the other robot, and we aimed to analyze the interaction with both robots. Nevertheless, employing eye tracking technology to measure visual fixations using the networked camera system and/or a head-mounted eye tracker is definitely one of the possible next steps. Another limitation to consider is the small sample size and the short-term exposure with the GEAR system that does not allow for inferences of rehabilitative effects in the general pediatric population. As previously stated, this methodology paper provides information on the design and rationale behind the inclusion of the different components of the system while also offering some evidence on the feasibility of the approach.

Complex learning environments like GEAR promise greater benefits. At the same time, such complex environments are “noisy” in the sense that they allow for considerable uncertainty and restrict the designer’s control authority. This becomes one of the major challenges to robot automation in this application space. We need to build robots that are adaptive; robots that can actively engage in play activities; robots that automatically and safely interact with young children in natural, real-world complex environments, such as the home or school. The ultimate goal is to enable high-dosage pediatric rehabilitation in natural and complex environments that could take place outside the structured setup of an academic lab or clinic. We envision “smart” environments that are robot-assisted but not human-sterile. The intention is not for the automated CRI system to become the sole form of interaction for the young child. Instead, it is envisioned that judicious rehabilitation environment designs can serve as catalysts for peer-to-peer and other forms of (human) social interaction.

## Conclusion

GEAR is a novel robot-assisted learning environment designed for use in pediatric physical rehabilitation. Although the data reported in this methodology paper are preliminary, they demonstrate the potential of the GEAR system and training protocol to be used in future rehabilitation studies. Our future work will focus on testing a larger sample size of participants and of different diagnoses to evaluate the training effects due to long-term exposure to this new environment and create more advanced models for the different components.

## Data Availability

Please contact the authors for data requests. Data containing identifying information (i.e. images and video recordings containing human subjects) are not to be shared outside of the research team as approved by the University of Delaware’s Institutional Review Board.

## Abbreviations

BWS: Body Weight Support
CRI: Child Robot Interaction
GEAR: Grounded Early Adaptive Rehabilitation
IDT: Improved Dense Trajectories
MDP: Markov Decision Process
MIL: Multiple Instance Learning
MI-SVM: Multiple Instance Support Vector Machine
MV: Majority Voting
SVM: Support Vector Machine
SVM-MV: Support Vector Machine with Majority Voting
